# Identification of Novel and Recurrent Variants in *MYO15A* in Ashkenazi Jewish Patients With Autosomal Recessive Nonsyndromic Hearing Loss

**DOI:** 10.3389/fgene.2021.737782

**Published:** 2021-10-18

**Authors:** Kevin T. Booth, Yoel Hirsch, Anna C. Vardaro, Josef Ekstein, Devorah Yefet, Adina Quint, Tzvi Weiden, David P. Corey

**Affiliations:** ^1^ Department of Neurobiology, Harvard Medical School, Boston, MA, United States; ^2^ Dor Yeshorim, Committee for Prevention of Jewish Genetic Diseases, Brooklyn, NY, United States; ^3^ Dor Yeshorim, Committee for Prevention of Jewish Genetic Diseases, Jerusalem, Israel

**Keywords:** RNA-splicing, deafness, Ashkenazi Jewish, hearing loss, genotype-phenotype correlation, MYO15A

## Abstract

Hearing loss is a genetically and phenotypically heterogeneous disorder. The purpose of this study was to determine the genetic cause underlying hearing loss in four Ashkenazi Jewish families. We screened probands from each family using a combination of targeted mutation screening and exome sequencing to identifiy the genetic cause of hearing loss in each family. We identified four variants in *MYO15A*, two novel variants never previously linked to deafness (c.7212+5G>A and p.Leu2532ArgfsTer37) and two recurrent variants (p.Tyr2684His and p.Gly3287Gly). One family showed locus heterogeneity, segregrating two genetic forms of hearing loss. Mini-gene assays revealed the c.7212+5G>A variant results in abnormal splicing and is most likely a null allele. We show that families segregrating the p.Gly3287Gly variant show both inter and intra-familial phenotypic differences. These results add to the list of MYO15A deafness-causing variants, further confirm the pathogenicity of the p.Gly3287Gly variant and shed further light on the genetic etiology of hearing loss in the Ashkenazi Jewish population.

## Introduction

Myosins are a superfamily of actin-based motor proteins that play an essential role in a wide variety of cellular activity ranging from intracellular transport and signaling, cell migration, and adhesion to muscle contractions (Manor and Kachar, 2008; Coluccio, 2020a; Coluccio, 2020b). Pathogenic variants in the genes that encode myosin proteins have been linked to many diseases, including hearing loss (Friedman et al., 2020). Currently, pathogenic variants in six myosin genes (*MYO3A, MYO6, MYO7A, MYH14, MYH9,* and *MYO15A*) have been linked to human deafness (Lalwani et al., 2000; Walsh et al., 2002; Donaudy et al., 2004; Friedman et al., 2020).

It is well established that pathogenic variants in *MYO15A* underlie autosomal recessive nonsyndromic hearing loss at the DFNB3 locus (Rehman et al., 2016; Hirsch et al., 2021). To date, more than 370 variants in *MYO15A* have been linked to DFNB3-related hearing loss (https://deafnessvariationdatabase.org/) (Azaiez et al., 2018). This allelic diversity is strongly mimicked at the phenotypic level, with variable hearing loss thresholds (ranging from mild to profound), onset (congenital-to-postlingual), and stability (progressive vs. non-progressive) (Friedman et al., 2002; Rehman et al., 2016).

Here we add to the pathogenic allelic heterogeneity of *MYO15A* by implicating two new pathogenic variants in *MYO15A*, one splice-altering and one frameshift, that co-segregate with deafness in Ashkenazi Jewish families. We also shed light on the phenotypic variability associated with a common pathogenic synonymous variant in the Ashkenazi Jewish community.

## Methods

### Subjects

Four families of Ashkenazi Jewish ancestry, segregating autosomal recessive sensorineural hearing loss (ARSNHL), were ascertained for this study. Affected individuals underwent clinical examination and pure tone audiometry to measure hearing thresholds. After written informed consent to participate in this study was given, blood samples were obtained from all affected and unaffected family members, and genomic DNA was extracted.

### Variant Identification and Segregation Analysis

One proband from each family underwent genetic screening using a panel of 60 hearing-loss-causing mutations common in the Ashkenazi Jewish community, performed at Dor Yeshorim (http://doryeshorim.org), as described (Hirsch et al., 2021). Probands in which only one pathogenic allele was identified underwent direct sequencing of all exons and flanking introns of *MYO15A*. Probands negative for targeted mutation screening subsequently underwent Exome Sequencing (ES), bioinformatic analysis and variant prioritization, as described (Hirsch et al., 2021). Candidate variants were screened using gene-specific primers in all available family members to confirm segregation. All variants have been submitted to the Deafness Variation Database (https://deafnessvariationdatabase.org/) (Azaiez et al., 2018) for curation and incorporation.

### *In Silico* and *In Vitro* Splicing Analysis

Variants c.7212+5G>A and c.9861C>T impact on splicing were computationally predicted using MaxEnt (Wang et al., 2004) and Human Splicing Finder (HSF) (Desmet et al., 2009). *In vitro* mini-gene assays for splicing were carried out as described (Tompson and Young, 2017; Booth et al., 2018; Booth et al., 2019; Booth et al., 2020; Hirsch et al., 2021). Wildtype (WT) *MYO15A* (NM_016239.4) exons 34 and 35 were PCR amplified with gene-specific primers and ligated into the pre-constructed pSPL3 Exontrap vector. The c.7212+5G>A variant was introduced into the wildtype sequences using the Q5^®^ Site-Directed Mutagenesis Kit (New England Biolabs, Ipswich, MA), using the manufacturer’s protocol. Colonies were selected and grown, and plasmid DNA was harvested using the ZymoPure Plasmid II Miniprep and Midiprep Kits (ZYMO Research, Irvine, CA). Following sequence confirmation, WT and mutant mini-genes were transfected in triplicate into HEK293 and COS7 cells, and total RNA was extracted 48 h post-transfection using the Quick-RNA MiniPrep Plus kit (ZYMO Research, Irvine, CA). Using a random primer mix (ThermoFisher Scientific, Waltham, MA), cDNA was synthesized using AMV Reverse Transcriptase (New England Biolabs, Ipswich, MA). After PCR amplification, products were visualized on a 2% agarose gel, extracted, cloned, and then sequenced.

## Results

### Subjects, Variant Identification, Prioritization, and Segregation Analysis

**FIGURE 1:**
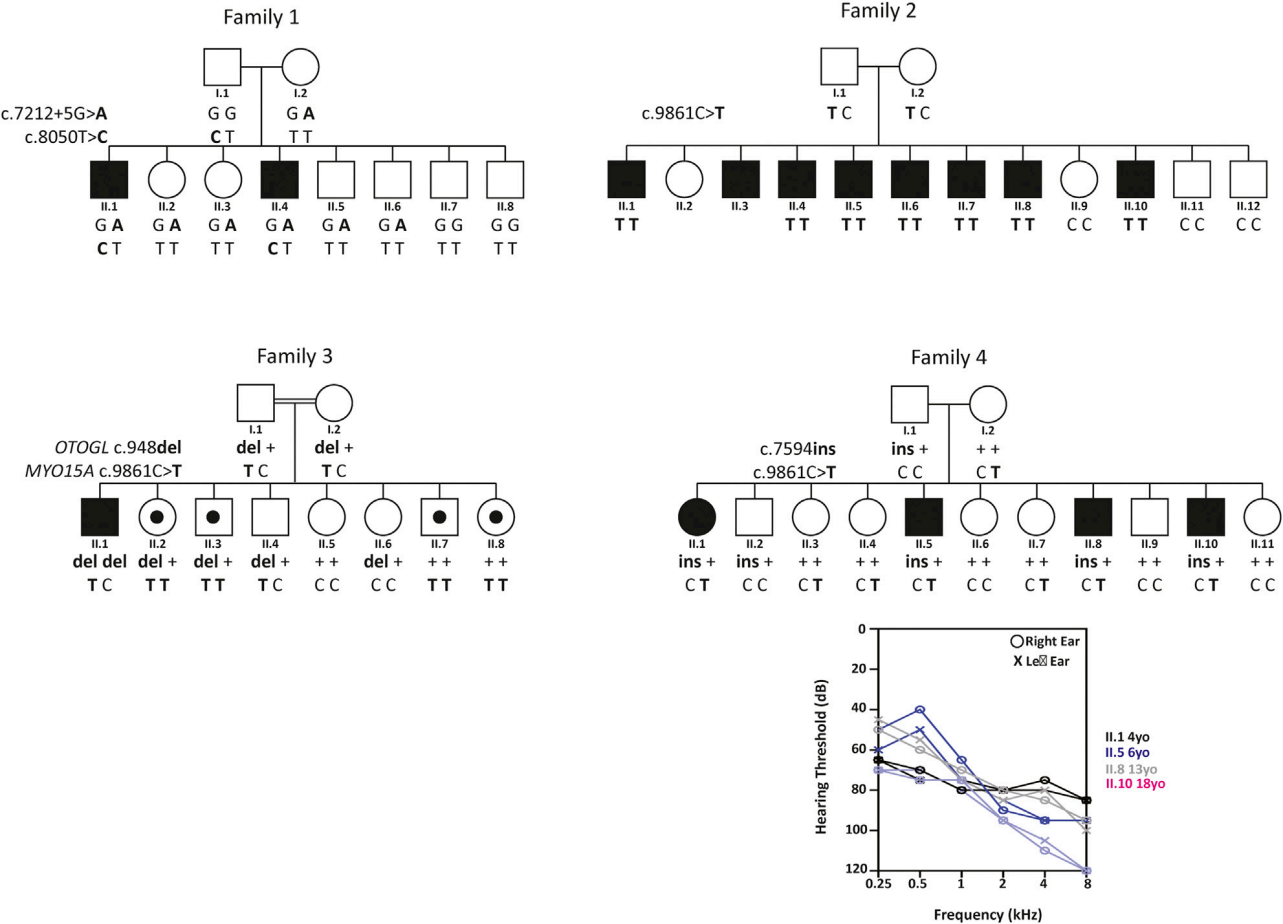
Pedigrees, corresponding genotypes for Families 1–4 and audiograms. Squares and circles represent males and females, respectively. Black fill indicates individuals with reported hearing loss, whereas no fill indicates unaffected individuals. Partially filled symbols represent individuals that are homozygous for the deafness-causing (c.9861C>T) p.Gly3287Gly variant but are reported to have normal hearing. Consanguinity is denoted by double lines. Genotypes of participating family members are shown below each symbol with bold indicating the mutant allele. Audiograms were obtained with air conduction with the frequency range of 250–8,000 Hz.

Affected individuals of Family 1 were born to non-consanguineous Ashkenazi Jewish parents. The proband (II.1) and his affected brother (II.4) have bilateral severe NSHL. The proband was diagnosed at 13 months and underwent cochlear implantation at 25 years old. His affected brother was diagnosed at 7 months and only uses hearing aids. The proband now 48 years old, reports a good outcome of his CI, and has impaired speech. Besides hearing loss, clinical evaluations were unremarkable. The proband underwent genetic testing using a high-throughput NGS panel for 60 known pathogenic variants that cause hearing loss, which revealed a heterozygous missense variant in *MYO15A* (c.8050T>C; p.Tyr2684His) (Table 1; Figure 1A). Direct sequencing of all coding exons of *MYO15A* and flanking introns revealed an ultra-rare second variant (c.7212+5G>A) in intron 35 (Table 1). Segregation analysis confirmed these two variants are in *trans* and segregate with the deafness in the family (Figure 1A).

**TABLE 1:** Variant table.

Variant				gnomAD			Conservation		Deleteriousness		Splicing			DVD	ClinVar	ACMG criterian	Final classification	References
Gene	Chr.pos	cDNA	Protein	Global MAF (%)	Max MAF (%)	Max pop	GERP	PhyloP	CADD	REVEL	MaxEnt		HSF					
											Diff	Alt						
*MYO15A*	17:18052899G>A	c.7212+5G>A (r.7212_7213ins7212+1_7212+16)	p.Ala2402fsTer29	0.0008	0.02	Ashk. J	C	C	22.9	—	4.9	1.36	Broken WT Donor Site	VUS	—	PVS1, PS3_P, PM2, PM3, PP1, PP3	P	This Study
*MYO15A*	17:18054544_18054545insGGGA	c.7594_7595insGGGA	p.Leu2532ArgfsTer37	0	0	—	—	—	—	—	—	—	—	—	—	PVS1, PM2,PM3,PP1_S	P	This Study
*MYO15A*	17:18057172T>C	c.8050T>C	p.Tyr2684His	0.006	0.07	Ashk. J	C	C	26.2	0.95	—	—	—	P	Con	PM3_S, PM2, PP1, PP3	LP	Cabanillas et al. (2018)
*MYO15A*	17:18069748C>T	c.9861C>T	p.Gly3287Gly=	0.017	0.41	Ashk. J	C	C	3.30	—	—	—	Significant alteration of ESE/ESS motifs	P	Con	PS4, PS3_P, PP1_S, PM3	P	Hirsch et al. (2021)
*OTOGL*	12:80633142del	c.948del	p.Leu316PhefsTer6	0.005	0.09	Ashk. J	—	—	32	—	—	—	—	P	P	PVS1, PM2, PM3	P	Azaiez et al. (2018)

Nucleotide numbering starting at the +1 position of transcripts NM_016239.4 and NM_001368062.1 for *MYO15A* and *OTOGL*, respectively. Minor allele frequency (MAF) from Genome Aggregation Database (gnomAD) version 2 (Havrilla et al., 2017). Deleteriousness was assessed using CADD, Combined Annotation Dependent Depletion (Kircher, 2014) and REVEL (Ioannidis et al., 2016). Variant impact on splicing was assessed using MaxEnt (Wang et al., 2004) and HSF, Human Splicing Finder (Desmet et al., 2009); ESE, Exonic Splicing Enhancer; ESS, Exonic Splicing Silencer. ACMG Criteria and Final Classification scoring based on Hearing loss specific ACMG guidelines (Oza et al., 2018).C, conserved; –, score not given or absent; Con, Conflicting entries; P, Pathogenic; LP, Likely Pathogenic; VUS, Variant of Uncertain Significance; _M, moderate; _P, supporting; _S, strong.

In Family 2, a homozygous synonymous variant in *MYO15A* (c.9861C>T; p.Gly3287Gly) was identified in the proband (II.1) and was found to segregate with the mild-to-moderate hearing loss in the family (Table 1; Figure 1B). The proband was initially diagnosed with mild, downsloping hearing loss at 12 years old and has reportedly progressed to moderate. His affected siblings are reported to have a similar hearing loss and progression. Age of diagnosis ranged considerably amongst the siblings, from 12 years old to 35 years old. Besides hearing loss, clinical evaluations in all affected individuals were unremarkable. Several affected individuals benefit from the use of hearing aids.

Family 3 is a large consanguineous family of Ashkenazi Jewish descent. The proband (II.1) was diagnosed at birth with bilateral, moderate, sensorineural hearing loss and had normal prenatal and postnatal clinical courses and neurodevelopment. At the time of testing, none of the seven siblings reported hearing loss. High- throughput NGS panel testing revealed the proband to be homozygous for a known pathogenic frameshift variant (c.948delG; p.Leu316PhefsTer5) in *OTOGL* and heterozygous for a known pathogenic synonymous variant (c.9861C>T; p.Gly3287Gly) in *MYO15A*. Segregation analysis revealed both unaffected parents are heterozygous for the *OTOGL* variant and the *MYO15A* variant (Figure 1D). Segregation in the extended family revealed four siblings with reported normal hearing to be homozygous for the p.Gly3287Gly variant in MYO15A (Figure 1C).

Affected individuals in Family 4 are reported to have prelingual nonsyndromic moderate-profound SNHL. The proband (II.1) underwent genetic testing using a high throughput NGS panel for 60 known pathogenic variants that cause hearing loss, which revealed a heterozygous synonymous variant in *MYO15A* (c.9861C>T; p.Gly3287Gly), but no second *MYO15A* pathogenic allele. Subsequently, the proband underwent ES, revealing a novel frameshift insertion (c.7594_7595insGGGA; p.Leu2532ArgfsTer37) in *MYO15A*. Segregation analysis confirmed these two variants are in trans and segregate with the deafness in the family (Figure 1D).

### Computational and *in Vitro* Splicing Analysis

**FIGURE 2:**
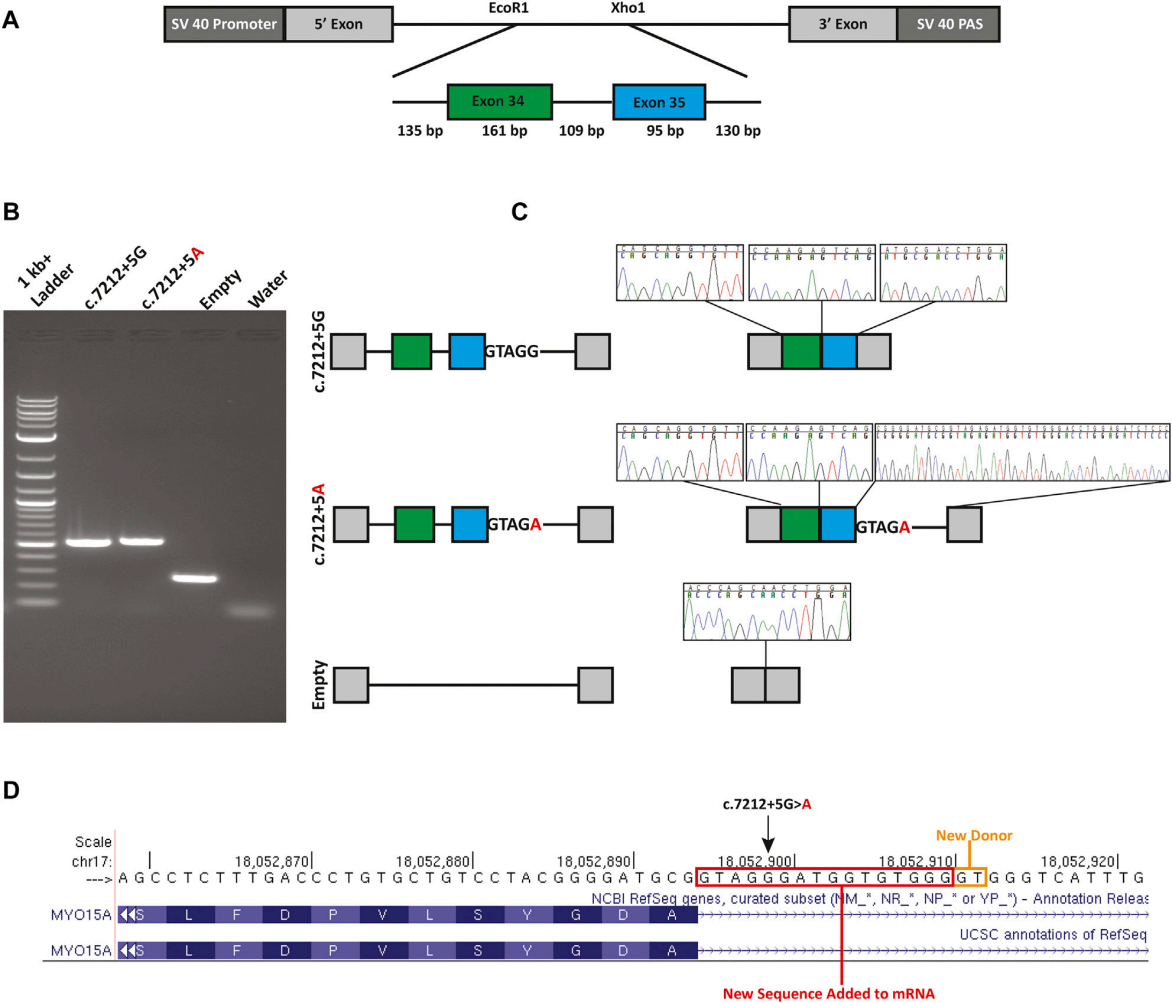
Mini-gene splicing assay. **(A)** Mini-gene design. Exons 34 and 35, the 109 bp intervening sequence and flanking intronic sequence of *MYO15A* were inserted into the EcoR1 and Xho1 site of the gene trap pSPL3 vector. **(B)** Gel electrophoresis of wildtype (c.7212+5G), mutant (c.7212+5A) and empty pSPL3 vector. The c.7212+5G>A variant causes the activation of a cryptic donor site. **(C)** Splicing schematic and chromatograms of sequenced spliced products. **(D)** Sequence chromatograms show the read through at each exon junction and sequence alignment shows the insertion due to the activation of a cryptic donor site. An orange box indicates the newly activated donor site and the red box highlights the retained intronic sequence.

The c.7212+5G>A variant is computationally predicted to alter the WT splicing and impact splicing (Table 1). To test improper splicing, we carried out *in vitro* mini-gene assays for splicing using the pSPL3 Exontrap vector. Visualization of the splicing products for WT and mutant mini-genes revealed a 263 bp band for the empty vector corresponding to the 5′ and 3′ native exons; a 519 bp product with WT sequence showing; and a 535 bp band for the mutant (Figure 2). Following gel extraction and cloning, eight colonies from the WT and 30 colonies from the mutant were picked and sequenced. All 8 WT colonies showed the expected wildtype splicing and inclusion of exons 34 and 35. Of the 30 colonies picked from the mutant, 3 colonies (10%) showed WT splicing but 27 colonies (90%) showed the activation of a new donor site (chr17:18052911-18052912) and the retention of 16 nucleotides in intron 35. Retention of 16 nucleotides in the *MYO15A* mRNA would result in a frameshift (c.7212_7213insGTAGGGATGGTGTGGG; p.Ala2402fsTer29) and the introduction of a termination codon in exon 36.

## Discussion

In this study, we used a combination of ethnicity-specific mutation screening and Exome Sequencing to implicate *MYO15A* and *OTOGL* as the causal genes underlying ARNSHL in four families of Ashkenazi Jewish descent. Of the variants identified, two affect RNA splicing, two are frameshifts, and one is a previously described pathogenic missense variant (Table 1). All affected individuals from families with reported consanguinity are homozygotes for the variants.

In Family 1, we initially identified a heterozygous previously described pathogenic variant (c.8050C>T; p.Tyr2684His) in *MYO15A* using a targeted variant panel. Subsequent ES revealed an ultra-rare variant (c.7212+5G>A) in intron 35 of *MYO15A*. The G to A transition alters the highly conserved canonical +5 guanine fundamental for the spliceosome protein U1 to bind to the 3’ end of an exon (Baralle and Baralle, 2005; Park and Cartegni, 2017). Using a mini-gene splicing assay we showed the G to A transition alters wildtype splicing via the loss of the canonical donor site and the activation of a new cryptic donor site. The use of this donor site adds 16 nucleotides to the reading frame of *MYO15A*, resulting in a frameshift and a new termination codon in exon 36 out of 66. Since this new termination codon is more than 50 base pairs from the last exon-exon junction, it is expected that this mutant transcript is subjected to nonsense-mediated decay (NMD) (Hentze and Kulozik, 1999; He and Jacobson, 2015) and is a null allele. It is possible that other mutant transcripts *in vivo* are created and were not detected due to the design of the mini-gene. In our analysis of the spliced products of the mutant mini-gene, three colonies (10%) out of the 30 colonies analyzed showed a wildtype splicing pattern. While this is only an approximation for correctly spliced transcripts, other methods such as RT-qPCR, would provide a more accurate quantification of the correctly spliced transcripts.

The pathogenic synonymous variant c.9861C>T (p.Gly3287Gly) in MYO15A was identified in three of the four families in this study. Affected individuals in Family 2 were homozygous for the variant. These individuals report a progressive post-lingual hearing loss, with a reported age of onset between 12 and 35 years old. Whereas affected individuals in Family 4 are compound heterozygous for p.Gly3287Gly and the novel frameshift c.7594_7595insGGGA (p.Leu2532ArgfsTer37), they are reported to have prelingual moderate-to-profound HL. Given its location in the gene, we expect the c.7594_7595insGGGA mutation to cause NMD (Hentze and Kulozik, 1999; He and Jacobson, 2015) and to be a null allele.

Finally, Family 3 had two pathogenic variants segregating in the family. The proband is homozygous for a frameshift variant (c.948delG; p.Leu316PhefsTer5) in *OTOGL*. This individual is also heterozygous for the p.Gly3287Gly variant in MYO15A. Segregation analysis for both variants revealed four reportedly unaffected siblings (II.2, II.3, II.7, and II.8) who are homozygous for the p.Gly3287Gly variant. At the time of ascertainment, the ages of II.2, II.3, II.7, and II.8 ranged from 3 years old to 12 years old. These individuals have been referred to an audiologist and otolaryngologist for further clinical evaluation. Family 3 showcases the complexities and intricacies associated with elucidating the genetic underpinning of disorders that are both genetically and phenotypically heterogeneous, such as with hearing loss. It also highlights the importance of carrier screening in siblings, who are at risk of developing the milder and later onsets of hearing loss.

The underlying cause of the phenotypic variability associated with the p.Gly3287Gly is currently unknown. Based on the data reported here and in previously published families (Hirsch et al., 2021), when the p.Gly3287Gly allele is in trans with a LOF allele, the HL has an earlier onset and is more severe. While in Family 2 the age of onset is reported to range from 12 to 35, it is possible that the age of onset was similar in all affected individuals, but was missed due to its mild nature. It is also possible that inter- and intra-familial differences reported in individuals who are homozygous for the variant may correlate with the leakiness of the splicing (Caminsky et al., 2014; Lombardi et al., 2021), wherein the more mutant splicing, the more severe the hearing impairment.

With a minor allele frequency of ∼0.5% in the Ashkenazi Jewish population, the p.Gly3287Gly allele represents a major contributor to HL in this population. A natural history study and further molecular work is needed to understand the exact cause of the reported phenotypic variability. Together this knowledge will improve patient care and clinical decision making. This knowledge will also be vital for any future gene therapies targeting *MYO15A* related hearing loss or the p.Gly3287Gly variant specifically.

In summary, we have identified two novel loss of function variants (one splice-altering and one frameshift) in *MYO15A* that cause ARNSHL in the Ashkenazi Jewish population. We provide more genetic support for the pathogenicity of the p.Gly3287Gly variant and illustrate that this variant exhibits both inter- and intra-familial phenotypic differences. Finally, our report highlights the importance of carrier screening and segregation analysis as it can identify individuals that will develop or have milder forms of disease.

## Data Availability

The datasets presented in this study can be found in online repositories. The names of the repository/repositories and accession number(s) can be found below: https://deafnessvariationdatabase.org/, DVD_001_KTB
